# Regression-based determination of cumin seed as a natural growth promoter in japanese quail

**DOI:** 10.1016/j.psj.2025.105881

**Published:** 2025-09-22

**Authors:** Mehran Mehri, Mahmoud Ghazaghi, Morteza Asghari-Moghadam, Hamid-Reza Behboodi, Mohammad Rokouei

**Affiliations:** a Department of Animal Sciences, Faculty of Agriculture, University of Zabol; Sistan 98613-35856, Iran; b Department of Animal and Poultry Physiology, Faculty of Animal Science, Gorgan University of Agricultural Sciences and Natural Resources; Gorgan 49138-15739, Iran

**Keywords:** Breasy meat yield, Cumin seed, Japanese quail, Performance, Regression

## Abstract

This study evaluated the effects of dietary cumin seed (CS) supplementation on the growth performance and carcass traits of Japanese quail chicks from 7 to 28 days of age. A total of 375 quail chicks were randomly assigned to five dietary treatments consisting of 0.0 (control), 10, 20, 30, and 40 g/kg CS, with five replicates per treatment in a completely randomized design. Results revealed that feed intake tended to be decreased by increasing CS (*P* = 0.133), whereas body weight gain increased linearly with CS inclusion, showing significant improvement compared with control (*P* = 0.017). Gain to feed ratio (G:F) exhibited a significant quadratic response, but overall differences among treatments were not significant (*P* = 0.117). Carcass traits were generally not influenced by CS, although breast meat yield tended to improve with increasing CS levels (*P* = 0.073). Regression analyses using various polynomial and broken-line models estimated the optimum dietary cumin seed inclusion to range between 21 and 35 g/kg diet for maximizing growth performance and feed efficiency. Breast meat yield optimization was predicted at approximately 25 to 29 g/kg. In conclusion, dietary supplementation of cumin seed can enhance growth performance in Japanese quail, with an optimal inclusion level estimated near 25 to 35 g/kg of diet. These findings provide guidance for using cumin seed as a natural growth promoter to improve productive efficiency in quail production.

## Introduction

Rearing practices in poultry nutrition increasingly emphasize natural feed additives to enhance productivity, animal health, and product quality while minimizing chemical residues and antibiotic use (Abd El-Ghany, 2020). Among various phytogenic compounds, cumin seed (*Cuminum cyminum* L.) has gained attention due to its bioactive constituents with multifaceted benefits, including growth promotion, antioxidant, antimicrobial, and digestive enhancement properties (Patil et al., 2017; Yitbarek, 2015).

Cumin seed contains essential oils rich in compounds such as cuminaldehyde, flavonoids, and phenolic acids, which exhibit antioxidant and anti-inflammatory activities, thereby potentially improving gut health and metabolic efficiency in poultry (Alimohamadi et al., 2014; Kikusato, 2021). Cumin and its derivatives also appear to encourage the release of digestive enzymes, such as amylase, protease, and lipase, which could boost nutrient use and feed conversion ratio (Keshamma, 2015; Milan et al., 2008).

Previous studies investigating the effects of cumin supplementation in poultry diets have yielded promising but sometimes inconsistent results. Berrama et al. (2017) found that cumin supplementation (20 g/kg) didn't significantly affect growth in heat-stressed chickens, but did improve feed efficiency. Conversely, Essien et al. (2024) demonstrated clear improvements in growth performance and carcass quality in broilers fed diets containing higher levels of cumin seed powder, without adverse effects on organ health. Similarly, Sumabala et al. (2021) documented improvements in feed efficiency and weight gain in quails supplemented with green cumin. Despite these findings, optimal dietary inclusion levels of cumin seeds remain unclear, and the specific effects on quail performance and carcass characteristics have not been thoroughly quantified. Moreover, advanced regression-based modeling provides powerful tools to estimate precise nutrient or additive requirements, facilitating more targeted feeding strategies for enhanced productivity (Ghazaghi et al., 2024).

Therefore, we hypothesized that supplementing quail diets with graded levels of cumin seed would dose-dependently improve growth performance (body weight gain and feed conversion ratio) and enhance carcass characteristics. The present study aimed to evaluate the effects of graded levels of cumin seed supplementation on growth performance and carcass traits in quail chicks, with a focus on the critical growth period up to 28 days of age, and to apply regression models to estimate the optimum dietary inclusion rate. Understanding these effects will contribute to developing natural growth-promoting strategies in quail production, aligning with the increasing demand for sustainable and residue-free poultry products.

## Materials and methods

### Ethics statement

All experimental procedures followed the guidelines of the Iranian Council of Animal Care and were approved by the Research Animal Ethics Committee of the University of Zabol (Approval No. AEUOZ-2012| JQ-2014-CS).

### Birds and experimental diets

A feeding trial was conducted to evaluate the effects of dietary cumin seed (CS) supplementation on Japanese quails from 7 to 28 days of age. From hatch to day 6, all birds received a standard starter diet formulated to meet or exceed NRC (1994) nutrient requirements. On day 7, a total of 375 quail chicks of uniform body weight (8.23 ± 0.70 g) were weighed using a digital scale (Model ML3002E, Mettler Toledo, Greifensee, Switzerland) and randomly allotted to five dietary treatments. The treatments consisted of a control diet without CS and four experimental diets supplemented with 10 20, 30, or 40 g/kg CS. Each treatment had five replicate pens, with 15 birds per pen. Feed and water were offered *ad libitum* throughout the experimental period. The house temperature was monitored daily using a digital thermohydrometer (Model 608-H2, Testo SE & Co. KGaA, Titisee-Neustadt, Germany) and maintained at 29 ± 2.10°C and relative humidity averaged 60 ± 2.0 % at the beginning of the experiment and then gradually decreased by 2.5°C per week until the end of the trial. The calculated temperature–humidity index (THI) during the trial averaged 22.9 (range: 20.0–25.8), indicating that the birds were maintained under thermoneutral conditions. Birds were exposed to an 18 h light:6 h dark photoperiod.

### Performance measurements

Body weight and feed intake were recorded by pen at weekly intervals (days 7, 14, 21, and 28). Weight gain and feed conversion ratio were calculated for each period and for the overall experimental duration. Mortality was checked daily and used to adjust performance data when applicable.

### Carcass traits

At 28 days of age, two birds were randomly selected from each experimental pen for organ sampling and carcass evaluation. The selected birds were weighed, slaughtered, and the breast and thigh muscles were excised to determine their relative weights as a percentage of live body weight.

### Statistical analysis

All data were analyzed using the GLM procedure in SAS (2002), with pen averages as the experimental unit. Data normality was checked prior to analysis using the Shapiro–Wilk test. Linear and quadratic responses to dietary CS were evaluated, and optimal dietary CS was estimated using broken-line regression models according to Khosravi et al. (2016):

Quadratic polynomial (QP):

$$Y_{i}=\beta_{0}+\beta_{1}x_{i}+\beta_{2}x_{i}^{2}+\varepsilon_{i},$$

where Y_i_ is the response, *β_0_*: intercept. *β*_1_​ is the linear (first-order) effect, *β*_2_ is the curvature, and *ε*_i_​ is the error term. The predicted optimum response is $-\beta_{1}\;/\;2\beta_{2}$.

Two-slope broken line:

LL: Linear ascending (or descending)-linear descending (or ascending)
Y = L + U × (R – X) × (X < R) + V × (X – R) × (X > R)
QL: Quadratic ascending (or descending)-linear descending (or ascending):
Y = L + U × (R – X)^2^ × (X < R) + V × (X – R) × (X > R)
QQ: Quadratic ascending (or descending)- quadratic descending (or ascending)
Y = L + U × (R – X)^2^ × (X < R) + V × (X – R)^2^ × (X > R)
One-slope broken line:
LBL: Linear ascending (or descending)-plateau
Y = L + U × (R – X) × (X < R)
QBL: Quadratic ascending (or descending)-plateau
Y = L + U × (R – X)^2^ × (X < R)

where Y represents the bird's response; L denotes the asymptote for the first segment; U and V correspond to the slopes of the first and second segments, respectively, indicating an increasing or decreasing trend; and R represents the breakpoint, which is considered the available phosphorus (AP) requirement.

Model selection for each variable prioritized the highest R² and lowest *S*_y_.ₓ (residual standard deviation):

$$S_{y.x}=\;\sqrt{\frac{SS}{df}}$$

## Result and discussion

Table 1.

**Table 1:** Composition of the basal diet.

Ingredient	Percent
Corn	43.26
Soybean meal	31.90
Corn gluten meal	11.91
Sand	4.00
Wheat	2.89
Soybean oil	2.12
Di-calcium phosphate	0.72
Limestone	1.43
NaHCO_3_	0.49
L-Lysine HCl	0.39
DL-Methionine	0.26
Mineral premix^1^	0.25
Vitamin premix^2^	0.25
NaCl	0.01
L-Threonine	0.12
Nutrient specifications	
AME (kcal/kg)	2900
CP ( %)	25.0
Methionine ( %)	0.75
Lysine ( %)	1.38
Threonine ( %)	1.03
Ca ( %)	0.80
Available P ( %)	0.30
DEB (mEq/kg)^3^	250

^a^Mineral premix provided per kilogram of diet: Mn (from MnSO4·H2O), 65 mg; Zn (from ZnO), 55 mg; Fe (from FeSO4·7H2O), 50 mg; Cu (from CuSO4·5H2O), 8 mg; I [from Ca (IO3)2·H2O], 1.8 mg; Se, 0.30 mg; Co (from Co2O3), 0.20 mg; Mo, 0.16 mg.

^b^Vitamin premix provided per kilogram of diet: vitamin A (from vitamin A acetate), 11,500 IU; cholecalciferol, 2,100 IU; vitamin E (from dl-α-tocopheryl acetate), 22 IU; vitamin B12, 0.60 mg; riboflavin, 4.4 mg; nicotinamide, 40 mg; calcium pantothenate, 35 mg; menadione (from menadione dimethylpyrimidinol), 1.50 mg; folic acid, 0.80 mg; thiamine, 3 mg; pyridoxine, 10 mg; biotin, 1 mg; choline chloride, 560 mg; ethoxyquin, 125 mg.

^c^Dietary Electrolyte Balance: represents dietary Na +K − Cl in mEq/kg of diet

The effects of CS in diet on performance and carcass traits of quail chicks were reported in Tables 2 and 3, respectively. Feed intake was not significantly affected across treatments (*P* = 0.133). The contrast of control *vs.* CS treatments was not significant (*P* = 0.101), and the linear effect was also non-significant; however, a significant quadratic effect was detected (*P* = 0.018). Body weight gain (G) showed a tendency toward significance among treatments (*P* = 0.081). The contrast of control *vs.* CS treatments was significant (*P* = 0.017), and a significant linear response was observed (*P* = 0.010), while the quadratic effect was not significant (*P* > 0.05). Gain:feed ratio was not different across treatments (*P* = 0.117). The contrast of control *vs.* CS treatments was not significant (*P* = 0.061), nor was the linear effect, but a significant quadratic effect was present (*P* = 0.029). Live weight was not significantly affected by dietary CS supplementation, with no differences for the control *vs.* CS comparison, linear effect, or quadratic effect. Similarly, thigh meat yield (TMY) was not influenced, with the control *vs.* CS contrast, linear, and quadratic effects, all being non-significant. Breast meat yield (BMY) was not significantly affected overall, nor by the control *vs.* CS comparison. A trend was observed for a linear effect (*P* = 0.108) and a quadratic effect (*P* = 0.073), although these did not reach statistical significance. Overall, cumin seed supplementation reduced feed intake by up to 4.7 % (20 g/kg) compared with the control, while body weight gain increased by approximately 2.7–2.9 % at 20–40 g/kg. The gain:feed ratio improved consistently, with the greatest enhancement of about 7.2 % at 20 g/kg, and remained higher than control at 30 and 40 g/kg. Live weight showed only minimal variation (–1.8 to +0.1 %), and thigh meat yield was largely unaffected (–2.4 to +0.6 %). Breast meat yield increased slightly at 10–30 g/kg (+0.7 to +1.4 %) but declined by 5.3 % at 40 g/kg. Overall, cumin seed inclusion up to 30 g/kg reduced feed intake while improving growth performance and feed efficiency, without adverse effects on carcass traits.

**Table 2:** Effects of different levels of cumin seed (CS; g/kg) on the feed intake (FI), body weight gain (G), gain:feed, live weight (LW), thigh meat yield (TMY), and breast meat yield (BMY) of growing Japanese quail from 7 to 28 d of age.

Response	CS (g/kg)					SEM	Probabilities			
	0.0	10	20	30	40		Treatment	Control vs. CS	Linear	Quadratic
FI (g/b)	385	378	367	370	383	5.48	0.133	0.101	0.506	0.018
G (g/b)	122.7	124.1	126.0	126.0	126.2	0.95	0.081	0.017	0.010	0.261
Gain:feed	0.320	0.328	0.343	0.340	0.328	0.01	0.117	0.061	0.192	0.029
LW (g)	149.3	146.6	149.3	148.6	149.4	2.26	0.885	0.749	0.746	0.662
TMY ( %)	16.7	16.6	16.8	16.3	16.4	0.41	0.901	0.754	0.526	0.876
BMY ( %)	28.1	28.4	28.5	28.3	26.6	0.59	0.181	0.769	0.108	0.073

SEM: Standard error of mean

**Fig. 1:**
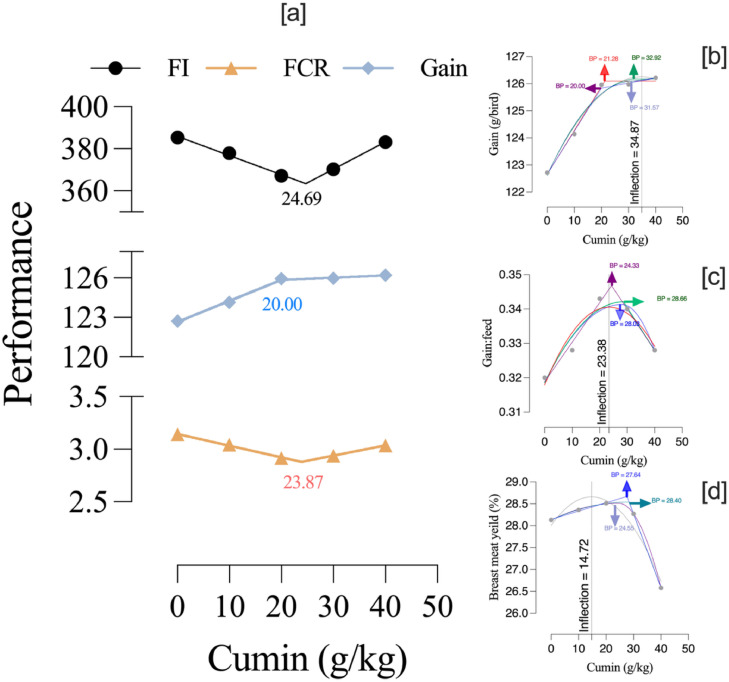
Effect of dietary cumin supplementation on quail performance indices. Panel [a] depicts the two-slope broken-line regressions fitted for feed intake (FI), feed conversion ratio (FCR), and body weight gain (Gain). Panels [b], [c], and [d] present the optimization of Gain, gain-to-feed ratio, and breast meat yield, respectively, using quadratic polynomial (QP) and various broken-line (one-slope and two-slope) models. The inflection point estimated from the QP model represents the maximum response point for each performance parameter.

The regression analyses provided different estimates of the optimum cumin seed level depending on the model used. Cumin supplementation showed that the breakpoint for feed intake occurred at 24.69 g/kg, for gain at 20.00 g/kg, and for feed conversion ratio at 23.87 g/kg (Fig. 1a). The LBL model yielded the most conservative estimate, with an optimum of 21.3 g/kg (95 % CI: 14.9–28.3) and the best statistical fit (R² = 0.994, RMSE = 0.118). In contrast, the QP equation predicted a higher requirement at 34.9 g/kg, although with lower fit accuracy (R² = 0.972, RMSE = 0.254). The QBL and two-slope models provided intermediate optima, ranging between 31.6 and 32.9 g/kg, with R² values of 0.973–0.974 and RMSE around 0.246–0.249, all of which passed normality homoscedasticity tests. Collectively, these results indicate that while the LBL model statistically describes the data with highest precision, the quadratic-based models consistently predict a higher dietary requirement, suggesting the optimum cumin seed inclusion lies closer to 31–35 g/kg depending on the chosen model (Fig. 1b). For the feed efficiency trait (G:F), the models provided different optimum estimates and levels of fit. The two-slope LL model estimated an optimum of 24.3 g/kg with a high accuracy (R² = 0.977, RMSE = 0.00143), and residuals showed no significant deviation from assumptions. The QP model predicted a slightly lower optimum of 23.4 g/kg but with weaker fit (R² = 0.883, RMSE = 0.00325). The two-slope QL and QQ yielded higher estimates of 28.7 and 28.0 g/kg, respectively, though with lower adjusted R² values (0.66–0.68) and RMSE around 0.0027–0.0028. All models passed normality and homoscedasticity tests. Overall, while the LL model demonstrated the best statistical performance and a more conservative optimum (∼24 units), quadratic approaches suggested higher requirements (∼28 units), indicating that the true dietary level for maximizing G:F likely lies between these ranges depending on the chosen model. For the BMY, all models predicted high accuracy but with differences in the estimated optimum cumin seed level. The two-slope LL model estimated the optimum at 27.6 g/kg with very strong fit (R² = 0.9996, RMSE = 0.0163). The two-slope QL model predicted a slightly higher optimum of 28.4 g/kg (95 % CI: 27.3–29.4) and exhibited nearly perfect fit (R² = 1.000, RMSE = 0.0047). Its variant, two-slope QQ, produced a lower estimate of 24.6 g/kg, though still with high accuracy (R² = 0.9998, RMSE = 0.0102). In contrast, the QP model gave the lowest estimate at 14.7 g/kg and had the weakest statistical performance among the models (R² = 0.921, RMSE = 0.221). All models passed normality and homoscedasticity tests, indicating unbiased residuals. While the QP model underestimated the requirement, the broken-line models consistently supported an optimum range around 27–29 g/kg for maximizing BMY.

This enhancement in growth and feed efficiency is likely attributable to the bioactive compounds in cumin, such as cuminaldehyde, flavonoids, and phenolic acids (Patil et al., 2017). These compounds are known to stimulate the secretion of digestive enzymes, including amylase, protease, and lipase, which improves the digestion and absorption of nutrients (Milan et al., 2008; Sumabala et al., 2021). Furthermore, cumin can promote bile secretion, which is crucial for fat emulsification and absorption (Keshamma, 2015). By maximizing nutrient utilization from the feed, the birds can achieve greater weight gain from the same or even slightly less feed, thereby improving the gain:feed ratio. Feed intake was largely unaffected, which aligns with some previous studies. For instance, Berrama et al. (2017) found that supplementing basal diets with 20 g/kg cumin did not significantly alter feed consumption or final body weight in chickens, though feed conversion ratio tended to improve. Conversely, Essien et al. (2024) reported enhanced growth and carcass quality in broilers supplemented with up to 45 g/kg cumin seed powder, without negative impact on internal organ weights. Sumabala et al. (2021) observed improved feed efficiency in quails supplemented with green cumin, attributed mainly to enhanced nutrient utilization rather than reduced intake. The lack of increased feed intake, despite higher weight gain, reinforces the hypothesis that cumin's primary effect is on improving digestive efficiency (Milan et al., 2008). When birds extract more nutrients from each gram of feed, their energy and nutrient requirements can be met with a lower volume of intake, potentially leading to earlier satiety. Additionally, the aromatic properties inherent to phytogenic compounds like cumin may influence diet palatability (Yitbarek, 2015), contributing to the slight reduction in consumption observed. The tendency toward improved weight gain in this study, supported by the significant linear increase, may be explained by several physiological effects of cumin. Enhanced digestive enzyme secretion, including amylase, protease, lipase, and phytase (Milan et al., 2008; Sumabala et al., 2021), and stimulation of bile secretion accelerates digestion and nutrient uptake (Keshamma, 2015) resulted in growth acceleration without increasing feed intake.

The lack of significant changes in live weight, TMY, and BMY is consistent with Berrama et al. (2017) and indicates that cumin supplementation at tested levels does not adversely affect carcass composition. Although BMY showed trends toward improvement, these did not reach statistical significance, suggesting that higher or more prolonged supplementation might be needed to elicit pronounced effects on carcass traits, as suggested in other studies (Alimohamadi et al., 2014). Beyond digestive enzyme stimulation, cumin's bioactive compounds, including flavonoids, phenolic acids, and essential oils, confer antioxidant, antimicrobial, and anti-inflammatory effects (Alimohamadi et al., 2014; Patil et al., 2017). These properties may improve gut health and nutrient absorption, reduce oxidative stress, and enhance immunity, thereby supporting better growth and feed efficiency (Alimohamadi et al., 2014). Recent advancements using nanotechnology to encapsulate cumin essential oils have demonstrated enhanced antimicrobial and antioxidant potential, further improving gut microbial balance and nutrient utilization (Abdelli et al., 2021; Jabbar et al., 2024). Such technologies hold promise for boosting the efficacy of phytogenic feed additives like cumin in poultry production.

Determining the precise optimum level of CS inclusion is critical for practical feed formulation. Our regression models yielded slightly varying estimates, but collectively suggest that optimal inclusion levels lie approximately between 21 and 35 g/kg, depending on the performance criterion. The broken-line models, especially the two-slope broken-line, provided conservative and precise estimations, favored in nutritional studies for breakpoint detection (Ahmadi et al., 2025; Ghazaghi et al., 2024; Pourmollaei et al., 2024). In the present study, regression analysis provided valuable insights into the dose–response relationship of the additive. While quadratic models offered higher optimum estimates with slightly poorer fits, they suggested potential for incremental benefits beyond the breakpoint. This highlights that the choice of analytical approach can influence interpretation of optimum inclusion levels. Conventional ANOVA with mean separation across discrete treatments is useful for detecting differences, but it may not fully capture the continuous nature of physiological responses. In contrast, regression modeling allowed us to better describe the biological trends observed in our data and to estimate practical dietary optima with greater precision.

Our findings indicate that cumin seed can be included in quail diets as a natural growth promoter, with an optimal level of approximately 24–29 g/kg. This level improves growth performance and feed efficiency without negative effects on carcass traits, providing a practical guideline for quail production.

## CRediT authorship contribution statement

**Mehran Mehri:** Writing – review & editing, Writing – original draft, Formal analysis, Conceptualization. **Mahmoud Ghazaghi:** Writing – review & editing, Resources, Funding acquisition. **Morteza Asghari-Moghadam:** Writing – review & editing, Resources, Funding acquisition, Data curation. **Hamid-Reza Behboodi:** Resources, Validation, Writing – review & editing. **Mohammad Rokouei:** Writing – review & editing, Visualization, Validation, Software, Data curation.

## Disclosures

The authors declare that they have no known competing financial interests or personal relationships that could have appeared to influence the work reported in this paper.
